# Simultaneous Stereo Matching and Confidence Estimation Network

**DOI:** 10.3390/jimaging10080198

**Published:** 2024-08-14

**Authors:** Tobias Schmähling, Tobias Müller, Jörg Eberhardt, Stefan Elser

**Affiliations:** 1 Institute for Photonic Systems Hochschule Ravensburg-Weingarten, University of Applied Sciences, Doggenriedstraße, 88250 Weingarten, Germany; tobias.mueller@rwu.de (T.M.); joerg.eberhardt@rwu.de (J.E.); 2 Institute for Artificial Intelligence Hochschule Ravensburg-Weingarten, University of Applied Sciences, Doggenriedstraße, 88250 Weingarten, Germany; stefan.elser@rwu.de

**Keywords:** stereo vision, confidence, multi-task learning, uncertainty

## Abstract

In this paper, we present a multi-task model that predicts disparities and confidence levels in deep stereo matching simultaneously. We do this by combining its successful model for each separate task and obtaining a multi-task model that can be trained with a proposed loss function. We show the advantages of this model compared to training and predicting disparity and confidence sequentially. This method enables an improvement of 15% to 30% in the area under the curve (AUC) metric when trained in parallel rather than sequentially. In addition, the effect of weighting the components in the loss function on the stereo and confidence performance is investigated. By improving the confidence estimate, the practicality of stereo estimators for creating distance images is increased.

## 1. Introduction

Stereo vision is a technology to determine the distance with two RGB cameras. A very high accuracy can be achieved by a precise correspondence search between the two images. Reconstructing the geometric configuration of a 3D environment is one of the fundamental and essential problems in computer vision fields [1]. The images of the two cameras are calibrated and rectified so that the corresponding points have the same *y*-value. Subsequently, for each pixel (*x*, *y*) in the right image, the corresponding pixel (*x* + *d*, *y*) in the left image is searched for. Once the pixel is found, the physical distance can be determined by geometry using the associated disparity value *d*. Any disparity value (*d*) can be converted into a distance value (*z*) using the following formula, given the focal length (*f*) and the base length (*b*) between the two cameras:

(1) $$\begin{array}{c} z=\frac{b\times f}{d} \end{array}$$

For a deeper theoretical insight, we recommend the book “Multiple View Geometry in Computer Vision” by Hartley and Zisserman [2]. The challenge is to find the right counterpart in the other image. Over the years, researchers have developed a variety of approaches to master the task of stereo matching. These methods have encompassed both handcrafted techniques [3,4,5,6] and machine learning-based approaches [7,8]. However, the complexity of the problem arises from several challenging factors, including reflective surfaces, textureless regions, regions with repeated patterns, occlusions [9,10,11], as well as photometric deformations resulting from variations in illumination and camera specifications [12,13]. However, an incorrect correspondence estimation can result in a large error in distance determination, making it difficult to use stereo vision in practice.

In response to these challenges, there are two different points of view for recognizing errors in stereo vision. The first is to consider a confidence interval, which indicates the probability that the result is correct. In the second, uncertainty can be considered as the expected error magnitude. Both views are equally valid; we use the confidence view, however, which is widely used in stereo vision [14]. Methods for estimating confidence have been developed in parallel with stereo algorithms. There are classical approaches [15,16,17] as well as those that employ neural networks for estimating the confidence values. However, due to the focus on confidence estimation, stereo vision algorithms based on neural networks are not used to evaluate the confidence methods. Today, deep end-to-end networks outperform classical stereo methods for deriving dense disparity maps, so confidence estimation should also be tested on stereo vision methods using neural networks rather than classical methods. Our motivation is to increase the reliability of stereo algorithms. We think that a joint consideration of stereo and confidence estimation could be a good step to achieve this. Therefore, in this paper, we present a neural network that combines the state of the art in stereo vision and the confidence estimation of stereo vision. This network is capable of identifying stereo disparity and the corresponding confidence maps.

The combined training results in a significant improvement in the confidence estimation compared to training the two parts separately. This is common practice in the use of confidence networks such as LAF-Net [18]. This method offers an advantage, as the component of the network employed to estimate the disparity values is trained in a way to allow for a subsequent, more accurate estimation of confidence. The network consists of already successfully tested network components from AANet [19] as the stereo part and from LAF-Net for the confidence estimation. The proposed network has high potential to advance the field of stereo vision and pave the way for more robust and reliable applications.

Our method is unable to overcome the known limitation of stereo caused by poor lighting or textless regions. However, knowing that the predicted values could be wrong is a significant advantage.

## 2. Related Works

This section provides an overview of the most important related work in the field of stereo vision and stereo confidence estimation.

### 2.1. Stereo Vision

In computer vision, stereo vision has made significant progress with the adoption of neural networks. Initially, traditional algorithms were partly replaced with learning methods to enhance the effectiveness of the widely used SGM (Semi-Global Matching) [6] algorithm. One approach to improve matching costs involves using neural networks to evaluate the matching score between a pair of image matches. Notable examples of such networks are SGM-Net [20] and SGM-Forest [21]. DNet [22] was one of the first approaches that used the 2D architecture of U-Net [23] for stereo vision inspired by the well-known segmentation networks. There are also later approaches [24,25] that use a 2D architecture. Other stereo networks take their inspiration from the classic stereo pipeline. Numerous works [26,27,28,29] used a 3D convolutional layer architecture. GC-Net [26] was one of the first approaches to use continuous stereo adaptation with 3D convolutional layers. These frameworks first map the images through a 2D convolution network to obtain dense representations of the features. A 3D cost volume is then constructed over the 2D feature maps, either by concatenation [26] or correlation [30]. After that, the cost volume is filtered through a series of 3D convolutional layers before being mapped to a point-wise depth estimate by a differentiable argument of the minimum (arg-min) operator. There are many variations of this approach, such as using a 3D stacked hourglass to process the cost volume [30] or developing new aggregation layers [31] to improve accuracy. Unlike classical filtering algorithms such as SGM, 3D convolution is a differentiable approximation. The neural networks outperformed traditional methods on datasets such as KITTI [32] and Scene Flow Datasets [22]. The primary drawback of 3D convolutions is their intensive memory and computing requirements. However, to address this issue, certain methods [19,31] have opted to entirely replace the commonly used 3D convolutions with new components, achieving faster inference speeds while maintaining comparable accuracy. One of the works [19] proposed using an intra-scale cost aggregation method based on sparse points with AANet. In addition, the traditional cross-scale cost aggregation algorithm is approximated with neural network layers to handle large texture-free regions. The recent methods try to reduce the impact of erroneous rectification in stereo vision. RAFT-Stereo [33] takes advantage of the iterative refinement of the RAFT optical flow network [34] to develop a network suitable for stereo matching. SR-Stereo [35] shows that the stepwise regression architecture has a better generalization performance than iteration-based methods.

Li et al. [36] propose a CREStereo network with an adaptive group correlation layer (AGCL) because it can happen that the matching points are not perfect in a line. This can be caused by the fact that the images in the real world are not perfectly rectified. In addition to constantly improving accuracy, new work is also endeavoring to further increase efficiency by replacing the costs of 3D CNN with new types of architecture. The primary drawback of 3D convolutions is their intensive memory and computing requirements. However, to address this issue, certain methods [19,31] have opted to entirely replace the commonly used 3D convolutions with new components, achieving faster inference speeds while maintaining comparable accuracy. One of the works [19] proposed using an intra-scale cost aggregation method based on sparse points with AANet. MobileStereoNet [37] uses 2D MobileNet-V2 [38] blocks, a combination of point-wise, depth-wise, and point-wise layers, and expands them to 3D for stereo vision applications to create an efficient stereo network. LightStereo [39] improves performance by focusing on the channel dimension of the 3D cost volume, using channel-boosted 2D CNNs with inverted residual blocks for efficient cost aggregation on resource-constrained devices.

### 2.2. Confidence Estimation

In parallel with the further development of stereo algorithms, methods for estimating the confidence in the estimated disparity maps are also advancing. Confidence methods attempt to improve classical stereo methods by identifying erroneous pixels. There are purely classical approaches [15,16,17,40], which were later combined by a random forest [41,42] to estimate the reliability of each pixel.

In later works, the Convolutional Neural Network (CNN) was also used to estimate confidence, but it must be distinguished which data the network receives as input. Some work only on the basis of the disparity map and the RGB image [43,44]; others [18,45], additionally, use the cost volume to estimate a confidence level. Because of these additional data, not all stereo methods can be used for evaluation, so classical stereo algorithms such as SGM or AD-Census are often used. Alternatively, neural networks are used, which are similar to classical methods. These neural networks [46] construct a cost volume by deriving a probability value for each disparity hypothesis. Therefore, the method is very similar to classical stereo methods and can easily be used for confidence estimation. The focus of the confidence methods is on a good confidence estimate, which is why the stereo performance is not so important. An optimal scenario includes the integration of an excellent stereo assessment together with a simultaneous confidence assessment. The work [14] by Poggi et al. is one of the first to apply confidence methods to end-to-end stereo networks. They determine the confidence with different methods from the results of the Guided Aggregation Net (GANet) [31]. GANet is a stereo network and uses a 3D architecture that generates a feature volume that resembles the usual cost volume. The similarity of this feature volume to the cost volume allows for the application of ready-to-exist confidence methods. They showed the effectiveness of confidence estimation through feature volume analysis using an end-to-end stereo network. However, for this, it is necessary that the stereo network has a feature volume that is as large as the resolution of the input. However, this is not the case for all modern stereo networks. For example, AANet uses only 1/3 of the input resolution mapped as feature volume. In this case, the stereo confidence method must be adjusted. Mehltretter [47] used a Bayesian Neural Network to predict both uncertainty and stereo vision based on the GCNet [26] stereo network. Chen et al. [48] used the Group-wise Correlation Stereo Network (GwcNet) [30] as a base stereo network and created an uncertainty estimation subnetwork that extracts information from the intermediate multi-resolution. The networks are trained with a new loss function based on KL divergence applied to obtained histograms.

## 3. Method

The objective of this work is to demonstrate how confidence estimation can be improved using a new multi-task network. The network is provided with a rectified image pair, and simultaneously estimates the disparity and the confidence value for each pixel. The network consists of two main components, each with a specific task. The first component handles the correspondence search and predicts the disparity, while the second component is responsible for the confidence estimation of the stereo results. These components are interconnected and allow simultaneous training. The information exchange between the components is unidirectional. This means that the output of certain layers of the stereo image component provides the input for the confidence estimate, but not the other way around.

### 3.1. Stereo Vision Component

The stereo component was based on the structure of AANet [19]. AANet aims to achieve high performance and high speed at the same time. Due to this good compromise, it is still comparable to new approaches and it is well suited for one of our applications; moreover, its clear architecture allows a very good connection to the confidential network.

In order to achieve high speed, the use of complex 3D CNNs was avoided. Instead, the authors employed a scale-internal cost aggregation based on sparse points, which resolves the well-known issue of edge-fattening at disparity discontinuities. Moreover, the traditional cross-scale cost aggregation algorithm was approximated by using a neural network architecture to handle large textural regions. These modules are lightweight and can be integrated into existing architectures, significantly increasing their speed while maintaining accuracy. In this network, different cost function volumes are generated and subsequently merged. The fast response time, while maintaining high accuracy, makes the networks interesting for practical applications.

### 3.2. Confidence Estimation Component

**Figure 1:**
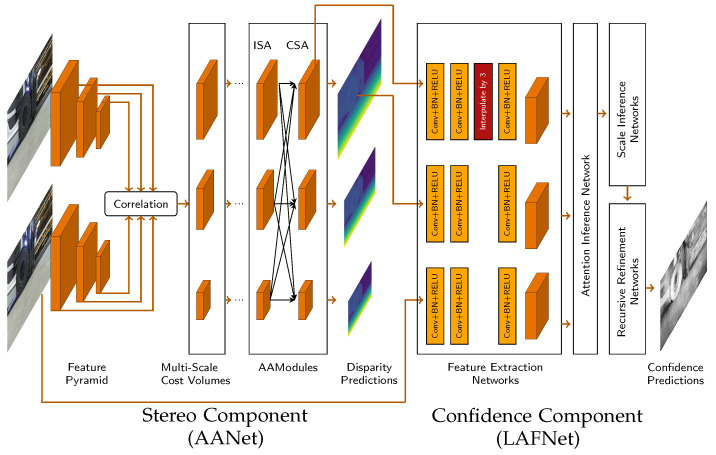
Proposed network structure, which consists of a combination of the AANet stereo network and the LAF-Net confidence network. A stereo pair is provided in the AANet to predict the disparity map. The output disparity in full resolution, the output tensor from the CSA with 1/3 resolution and the left input images serve as inputs for the LAF-Net component to predict the confidence. Due to the different resolutions, an interpolation layer must be included in the LAF-Net.

The confidence component was inspired by LAF-Net [18]. LAF-Net takes the disparity map from the stereo component, the cost volume, and the left color image as input, and produces a confidence map as output. However, instead of using all the total cost rates, only the *k* lowest values of the cost rates are used. These *k*-cost rates represent the most likely correct hypotheses with the highest probability. The value of *k* was set to 7, as it was found to perform effectively by the authors of the network. LAF-Net is specifically designed for classic stereo methods, where cost volume is the most important factor. Many stereo networks, including AANet, also provide some form of cost volume. In AANet, however, the cost volume is available in three different resolutions: 1/3, 1/6, and 1/12. For confidence estimation, the full resolution cost volume is required and adjustments need to be made. In addition, we did not use the cost volumes directly after correlation, but after the Cross-Scale Aggregation (CSA) layer. This approach gave the network more flexibility to optimize the cost variables for confidence estimation. The selection of a 1/3 resolution was motivated by its proximity to the desired resolution. However, to suit the specified resolution, the LAF-Net architecture required adjustments. To achieve this, an interpolation layer was introduced following the second convolution block while extracting features from the cost volume. The network with the two components and their connections to each other is shown in Figure 1.

### 3.3. Training

Our network was trained on the Scene Flow dataset, a large synthetic dataset that provides dense ground truth disparity maps. This dataset comprises more than 39,000 image pairs. Due to the high number of available data, real data were not used for training. Evaluation was also based on real data, without the need to fine-tune the network on real data. For the training, we created a loss function, which is a combination of the loss function of the stereo component ($\ell_{Stereo}$) and the LAF-Net component ($\ell_{Conf}$), defined as follows:

(2) $$\begin{array}{c} L=\ell_{Stereo}\left(D,D_{GT}\right)+\lambda \times \ell_{Conf}\left(C_{GT},C\right) \end{array}$$

where *D* and *C* are the predicted disparity and confidence maps. $D_{GT}$ and $C_{GT}$ are the respective ground truth values, which serve as labels. The new loss function is a weighted combination of the individual loss functions of the two network components. The weight is determined by the factor λ. The higher its value, the more emphasis is placed on optimizing the confidence estimate. The loss function of stereo is taken from AANet. It is calculated on the different disparity maps with different resolutions. A smooth L1 loss is generated for each disparity. A weighted sum is formed over the individual losses. The original implementation of the LAF-Net network used a classification loss. This means that the network should predict a value of 1 for a pixel if the error is below a threshold, otherwise it should predict 0. To achieve better coordination between the two loss functions, we used a regression loss function instead of a classification. This minimized the MSE between the $C_{GT}$ and *C*. It should be noted that the label $C_{GT}$ for this loss function can only be created after the prediction of the disparity map. The label $C_{GT}$ is determined as follows:

(3) $$\begin{array}{c} C_{GT}=e^{-|D_{GT}-D|} \end{array}$$

This ensures that the value is always between 0 and 1, where 1 corresponds to high confidence and a value close to 0 corresponds to low confidence.

## 4. Experiments

To demonstrate the benefits of training stereo and confidence in parallel, we initially conducted separate training for each network component, focusing on the Scene Flow datasets. This approach aligns with the traditional methodology employed by most confidence methods. By treating stereo and confidence networks as separate networks, we aimed to establish a comparative baseline.

To assess the benefits of simultaneous training under real conditions without the need for retraining, we evaluated our network using a combination of synthetic and real datasets. Specifically, we utilized Scene Flow [22], a synthetic dataset comprising over 39,000 stereo frames with a resolution of 960×540 pixels, for both training and testing our network. Additionally, we employed the widely used KITTI2015 [49] stereo dataset, to further test our network’s performance. Notably, we did not train our network but used the official training set of KITTI2015 for our evaluation, allowing for an unbiased evaluation. Furthermore, we incorporated the RWU3D [50] dataset, a novel dataset designed for the fusion of stereo and Time-of-Flight (ToF) sensors, capturing indoor industrial environments. The dataset includes images with a different resolution; in this work, the resolution 960×540 pixels was used. For testing purposes, we selected a subset of 32 images, excluding the calibration scene and scenes intended for characterizing the ToF camera.

### 4.1. Metrics

To evaluate our network, we employed a variety of evaluation metrics. In this regard, we adopted the evaluation methodology of LAF-Net, which exhibits subtle differences from the evaluation approach utilized in SEDNet. We prioritized the perspective of trust over uncertainty and have, therefore, opted for an evaluation approach. When determining the stereo error, the predicted disparity was taken into account and not the underlying physical distance, as the disparity represents the direct error of the network. The physical error of the distance also depends on the network, but also on the camera setting and the distance of the area under consideration, depending on which dataset is being evaluated

First, we used the training metrics. These included the Mean Squared Error (MSE) between the ground truth disparity and the predicted disparity, as well as the difference between the predicted confidence and the label for confidence. Second, we used additional evaluation metrics. We employed the Bad3 metric to evaluate the stereo part and used the area under the curve (AUC) to evaluate the confidence part.

#### 4.1.1. Bad3 (Bad Pixels Rate)

The Bad3 metric provides an assessment of the accuracy of our stereo model in estimating disparities. It measures the percentage of pixels for which the disparity error exceeds a threshold of 3. A lower Bad3 value indicates better performance, as it signifies a smaller proportion of pixels with large disparity errors. In the RWU3D dataset, an error of 3 pixels corresponds to an error of 0.034 m at a distance of 1 m.

#### 4.1.2. Area Under the Curve (AUC)

An optimal confidence algorithm produces a value for each pixel that is inverse to its error. The lower the error level, the higher the confidence should be. To evaluate the performance of each confidence measure in identifying correct matches, as outlined in [17], we sorted the pixels in a disparity map in descending order of confidence and calculated the error rate (Bad3) on sparse maps generated through iterative sampling (e.g., 5% of pixels at a time) from the density map. This means that first the error of 5% of pixels with the highest confidence was calculated. Next, the error of the 10% of pixels with the highest confidence was calculated, and so on. The plot of error rates produces a sparsification curve, which allowed us to quantitatively measure the effectiveness of the confidence measure through its AUC. The lower the AUC, the better the performance of the confidence measure. The optimal AUC is achieved when the confidence measure is able to identify all correct matches first and is equal to

(4) $$\begin{array}{c} AUC_{Opt}=\int_{1-\epsilon }^{1}\frac{x-(1-\epsilon )}{x}dx=\epsilon +\left(1-\epsilon \right)ln\left(1-\epsilon \right) \end{array}$$

with ϵ being the Bad3 computed over the disparity map. Looking at the ROC curve, one can tell how high the error is, for example, if 80% of the pixels with the highest confidence are observed.

### 4.2. Implementation Details

We implemented the network in PyTorch v1.13.1 and trained it with an Nvidia RTX 4090 GPU (Nvidia Corporation, Santa Clara, CA, USA). For training, we used 35,454 stereo pairs from the training set of the Scene Flow dataset. Before input, the raw images were randomly cropped to a size of 288×576. Adam [51] ($\beta_{1}=0.9$, $\beta_{2}=0.999$) was used as the optimizer. We started with a learning rate of 0.001, which was gradually halved every 10 epochs after 20 epochs. Each configuration was trained for 70 epochs, with a batch size of 4. Firstly, the stereo and confidence parts were trained sequentially, which corresponds to the results of the AANet and LAF-Net, whereby only the 1/3 resolution of the cost function could be used, as the AANet does not have a total cost function.

Secondly, the network was trained in parallel using the proposed loss function. The loss function was used with λ values of 5, 10, and 20. For each training configuration, the network was randomly initialized. It should be noted that the values for λ should be significantly greater than 1, as the values for stereo loss are much greater than those for the condensation loss function. This is due to the fact that the predicted value for the disparity was between 0 and 192, while the predicted value for confidence was only between 0 and 1.

## 5. Results

Table 1 presents the comparison metrics of different settings against SEDNEet. Overall, our networks outperform the SED network, primarily due to the use of AANet as the backbone for stereo matching, which already demonstrates superior stereo performance. However, it is noteworthy how much better our approach generalizes to real data compared to the SED network, without requiring re-training. The results in Table 1 also show that the combination of training confidence and stereo leads to an improved performance in terms of confidence. The results depend on the values used for λ in the loss function. With a small value of 5, the stereo results do not suffer, and the confidence performance is still better. Increasing the value of λ increases the MSE and Bad3 of the stereo, but decreases the MSE of confidence. This clear trend can be seen particularly well with Scene Flow. In principle, this observation can also be made with the other datasets, although this trend sometimes shows outliers. Several factors can explain this phenomenon. Overweighting the confidence components can result in overfitting, which may worsen performance on a new dataset. However, adjusting the confidence level slightly can prevent overfitting the stereo and improve, or at the very least maintain, the stereo score. Alternatively, the smaller real test dataset may have introduced some normal noise. To summarize, parallel training leads to a better overall result, which can be easily determined using the AUC value. When the stereo and confidence components are trained together, the AUC values are closer to the $AUC_{Opt}$ value than when they are trained separately. The $AUC_{Opt}$ value is the lower bound and is the value that can be theoretically achieved with optimal confidence estimation. The AUC value is influenced by both the stereo performance and the confidence performance. Therefore, this will be a good indication of which λ value is most appropriate. With the Scene Flow dataset, it is λ=5; with KITTI2015 and RWU3D, it is λ=10 where the lowest AUC is found. Therefore, we can deduce that an optimal value lies between 5 and 10.

**Figure 2:**
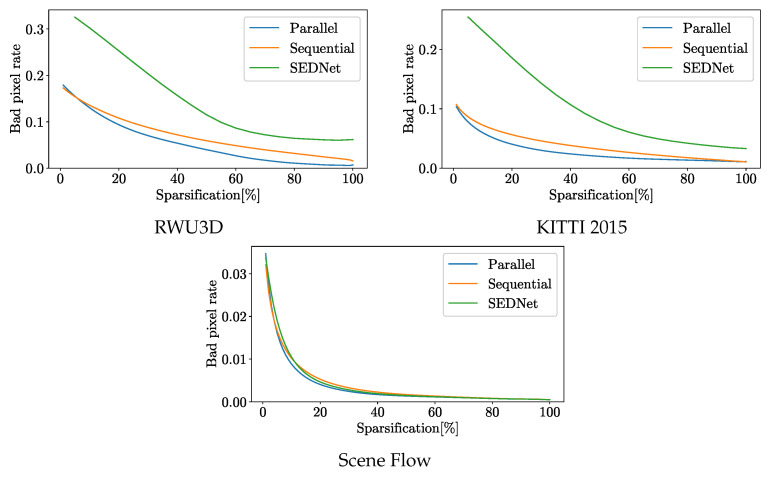
The sparsification curves show the rate of bad pixels at threshold 3 when a certain percentage of pixels with the lowest prediction confidence are excluded.

**Figure 3:**
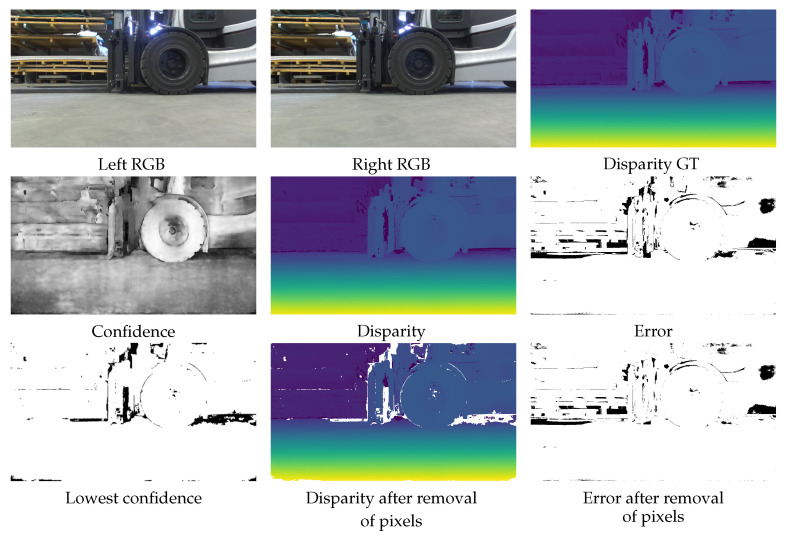
The example shows the left and right RGB input images and the ground truth disparity in the top row. The middle row shows the predicted confidence, the predicted disparity, and the error map of the disparity. In the error map, the black pixels have an error of more than 3 disparity values. Using the predicted confidence, 20% of the pixels with the lowest confidence were removed, resulting in the disparity map shown in the middle of the third row. The corresponding error map for this disparity map is shown next. In the disparity maps, yellow indicates high disparity (short distance), while dark blue signifies low disparity (long distance). It should be noted that the number of black pixels is now lower, indicating that the predicted confidence has removed pixels appropriately.

As mentioned above, the AUC is the most interesting value. So, let us look at the underlying sparsification curve shown in Figure 2. The curves show the advantage of parallel training with λ=10 even more clearly than the figures in the table. The curve shows a bad pixel rate when looking at the pixels with the highest confidence. The sparsification indicates the percentage of pixels with low confidence that are ignored. Especially for the two real test datasets RWU3D and KITTI2015, the improvement is even more visible than for the test data from Scene Flow. Therefore, we can assume that our training approach has even led to an increase in generalization ability or, at least, that no overfitting has taken place. In particular, the generalization to a new real dataset is evident when compared to SENet. This improvement is mainly due to the stereo component of the network, which has a strong influence on the AUC curve. Upon detailed examination of the sparsification curve Figure 2, it is possible to identify the bad pixel rates that are reached when only considering the pixels with the highest confidence values. By ignoring 20% of the low-confidence pixels identified through sequential training, the error rate of the remaining pixels in the KITTI2015 dataset is reduced to 0.05. With parallel training and an improved confidence estimation, this error rate could be further reduced to 0.035. This shows that, by masking pixels with low confidence, the total error of the overlaid pixels is smaller. However, for an application, it is important that the masked pixels are as evenly distributed as possible, which is the case with our confidence estimate, as can be seen in the example image in Figure 3. It is evident that the application of confidence estimation allows the removal of erroneous pixels. By establishing a threshold value of 0.3, any pixel with a confidence estimate lower than this threshold is excluded. This observation aligns with the AUC values, indicating that the filtered pixels are distributed throughout the image and do not signify a substantial loss of critical information.

**Figure 4:**
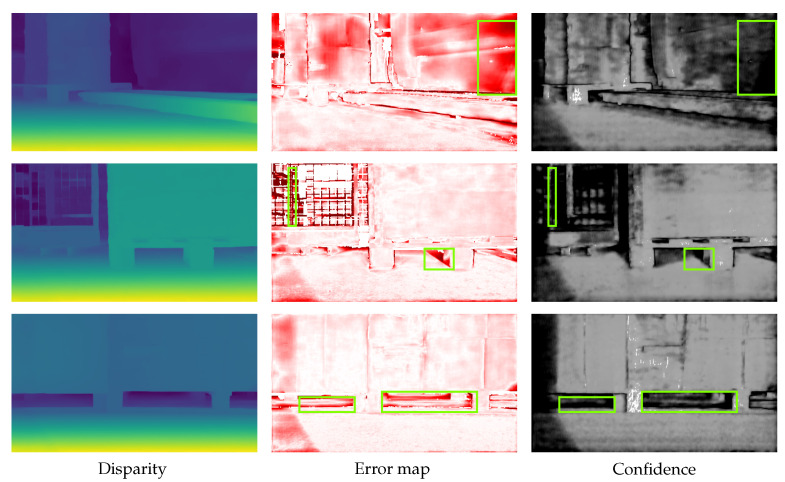
Example scene from the RWU3D dataset. In the left column, the disparity map is visualized, in the center is the corresponding error map, and on the right is the predicted confidence with white showing high confidence. In the disparity maps, yellow indicates high disparity (short distance), while dark blue signifies low disparity (long distance). The green rectangles show some areas where the confidence is correctly low, as there is also a larger error there.

The strong correlation between error and lower confidence prediction is evident in Figure 4, particularly within the highlighted example region. Regions with height errors often also have a lower confidence; this correlation can be used to obtain a higher confidence for the stereo image in the application.

### Computational Performance and Efficiency

The computational complexity of the proposed network is evaluated by measuring the time required for a single stereo and confidence prediction on a 960×540 image using an Nvidia RTX 4090 GPU. With a processing time of 98 ms, the network achieves a frame rate of more than 10 frames per second, which makes our network interesting for real-time applications. The stereo computer is the most complex of the networks and, by using the AANet’s computing power instead of other popular stereo networks such as GwcNet [30], a very high response time is still achieved. Additionally, the number of parameters shown in Table 2 is significantly lower than the number of parameters in SEDNet.

## 6. Conclusions

We have demonstrated the feasibility of combining deep stereo networks with a confidence estimation network. In contrast to the stereo techniques employed in evaluating LAF-Net, the stereo output produced by AANet exhibits minimal inconsistencies. However, our network successfully attains a robust confidence estimate. Training the stereo and confidence components simultaneously improves the overall results compared to training them sequentially. This can improve the AUC values by about 15% for the synthetic datasets. When using real datasets, an improvement of about 30% can be observed. It is therefore clear that parallel training offers benefits and is a potential form of multi-task learning. Additionally, our findings reveal that synergizing the training of stereo and confidence networks yields even more promising results. Through the judicious exclusion of pixels with lower confidence scores, we can generate stereo depth images that are notably more reliable. Applications in the field of same-vehicle driving could be interesting. Due to the higher filling reliability and the, nevertheless, light architecture, the approach can be used well on mobile devices. However, there is still scope for improvement to achieve the optimal confidence estimate. The proposed system could be adapted to some of the recently released stereo networks to achieve further improvements. LightStereo [39] could be a promising network for this approach.

## Figures and Tables

**Table 1:** Evaluation results with different training configurations for Scene Flow, KITTI2015, and RWU3D datasets. Parallel (λ) is our network with the different λ values (5, 10, and 20) for the loss function.

Network	MSE Disparity	MSE Confidence	Bad3 [%]	AUC	AUC_*opt*_
		Scene Flow			
SEDNet [48]	0.835	-	4.31	0.36	0.19
Sequential	0.680	0.0280	3.21	0.37	0.19
Parallel(5)	0.674	0.0236	3.34	0.32	0.20
Parallel(10)	0.695	0.0220	3.47	0.32	0.21
Parallel(20)	0.720	0.0203	3.71	0.33	0.21
		KITTI2015			
SEDNet [48]	10.828	-	35.33	9.55	4.61
Sequential	1.858	0.2705	10.70	3.71	1.23
Parallel(5)	1.878	0.2579	11.20	3.32	1.31
Parallel(10)	1.738	0.2921	10.36	2.76	1.20
Parallel(15)	1.852	0.2679	10.82	2.89	1.27
		RWU3D			
SEDNet [48]	11.835	-	39.07	13.47	7.10
Sequential	2.445	0.1547	17.30	6.77	2.32
Parallel(5)	2.681	0.0860	18.65	6.17	2.85
Parallel(10)	2.571	0.0824	17.89	5.22	2.46
Parallel(15)	2.613	0.0657	18.85	5.42	2.71

**Table 2:** Number of parameters.

Architecture	Parameters (M)
SEDNet	6.91
Our Network	
Stereo Component	3.93
Confidence Component	0.54
Total	4.47

## Data Availability

The data presented in this study are available on request from the corresponding author.

## Abbreviations

The following abbreviations are used in this manuscript:

ToF	Time of Flight
AUC	Area under the curve
Bad3	Bad Pixels Rate with threshold of 3
MSE	Mean Squared Error
CSA	Cross-Scale Aggregation
